# Leading modes of tropical Pacific subsurface ocean temperature and associations with two types of El Niño

**DOI:** 10.1038/srep42371

**Published:** 2017-02-17

**Authors:** Zhiyuan Zhang, Baohua Ren, Jianqiu Zheng

**Affiliations:** 1 School of Earth and Space Sciences, University of Science and Technology of China, Hefei, Anhui, China

## Abstract

Using empirical orthogonal function (EOF) analysis of the monthly tropical Pacific subsurface ocean temperature anomalies (SOTA) from 1979 to 2014, we detected three leading modes in the tropical Pacific subsurface temperature. The first mode has a dipole pattern, with warming in the eastern Pacific and cooling in the western Pacific, and is closely related to traditional El Niño. The second mode has a monopole pattern, with only warming in the central Pacific subsurface. The third mode has a zonal tripole pattern, with warming in the off-equatorial central Pacific and cooling in the far eastern Pacific and western Pacific. The second and third modes are both related to El Niño Modoki. Mode 1 is linked with a Kelvin wave that propagates from the central to the eastern Pacific and is induced by the anomalous westerlies that propagate from the western to the central Pacific. Mode 2 is also linked with a Kelvin wave that propagates from the western to the central Pacific induced by the enhancement of westerlies over the western Pacific. Mode 3 is linked with a Rossby wave that propagates from the central to the western Pacific driven by the anomalous easterlies over the eastern Pacific.

El Niño is an anomalous warming phenomenon in the central and eastern Pacific that has great influence on global climate. The existence of more than one type of El Niño in the tropical Pacific has been widely accepted, with many studies and names of the different types, such as dateline El Niño, El Niño Modoki, eastern Pacific (EP)/central Pacific (CP) El Niño and cold tongue (CT)/warm pool (WP) El Niño^1^,^2^,^3^,^4^,^5^,^6^,^7^,^8^. All these types of El Niño can be simply classified as traditional El Niño and El Niño Modoki (Pseudo El Niño). The greatest difference between these two types of El Niño is the distinct distribution of sea surface temperature anomalies (SSTA). The warming centre of traditional El Niño is in the eastern Pacific, whereas the warming centre of El Niño Modoki is located in the central Pacific^2^,^3^,^4^,^6^,^7^,^8^,^9^. In addition, El Niño Modoki has two cooling centres located in the western and eastern Pacific, respectively^3^,^4^. The anomalous zonal wind distributions associated with the two types of El Niño are also different. Traditional El Niño is dominated by strong westerlies over the central Pacific and only weak easterlies appear in the far eastern Pacific^9^. In contrast, significant westerlies and easterlies are found in the western and eastern Pacific, respectively, during El Niño Modoki^3^,^4^,^7^,^9^. The dynamical mechanisms of the two types of El Niño are also very different. Ashok *et al*.^3^ has suggested that traditional El Niño is associated with the equatorial Kelvin wave driven by the westerlies but El Niño Modoki is associated with the Kelvin and Rossby waves that are driven by the anomalous westerlies from the western Pacific and the easterlies from the eastern Pacific, respectively^3^.

Traditional El Niño is produced by the feedback from basin-wide thermocline variations. On the other hand, El Niño Modoki is generally considered to be a standing pattern with subsurface ocean temperature anomalies (SOTA) located in central Pacific^3^,^4^,^6^. The distributions and evolutions of the SOTA of the two types of El Niño are even more distinct than those of the SSTA^10^,^11^. Singular value decomposition (SVD) analysis was used to study the related modes in the subsurface of the two types of El Niño. A dipole pattern with warming in the eastern Pacific and cooling in the western Pacific and a zonal tripole pattern with warming in the central Pacific and cooling in the western and far eastern Pacific were found in the subsurface and are related to traditional El Niño and El Niño Modoki, respectively^11^. Another finding has suggested that three evolution patterns of El Niño Modoki exist that are determined by equatorial thermocline structures^12^. This scenario would indicate the presence of more than one pattern related to El Niño Modoki in the subsurface. Therefore, the tropical Pacific subsurface patterns need to be investigated independently. What are the spatial structures of the subsurface patterns? How are they related to traditional El Niño and El Niño Modoki? How do they evolve, and what are their possible mechanisms? This paper aims to answer these questions.

Many indices have been proposed to characterize El Niño^3^,^10^,^13^,^14^. Generally, the El Niño events can be characterized by NIÑO3.4^15^. However, this index cannot distinguish traditional El Niño from El Niño Modoki well. NIÑO3 and IEMI (improved El Niño Modoki index) have been proven to be better indices for characterizing traditional El Niño and El Niño Modoki, respectively^13^. In this paper, we adopt NIÑO3 and IEMI for two types of El Niño, respectively. NIÑO3 is defined as the monthly SSTA averaged over the region NIÑO3 (150W-90W, 5S-5N), and IEMI is defined as follows:


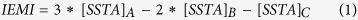


The brackets represent the averaged SSTA for regions A (165E-140W, 10S-10N), B (110W-70W, 15S-5N) and C (125E-145E, 10S-20N), respectively.

## Results

### Empirical orthogonal function (EOF) results and associations with traditional El Niño and El Niño Modoki

**Figure 1:**
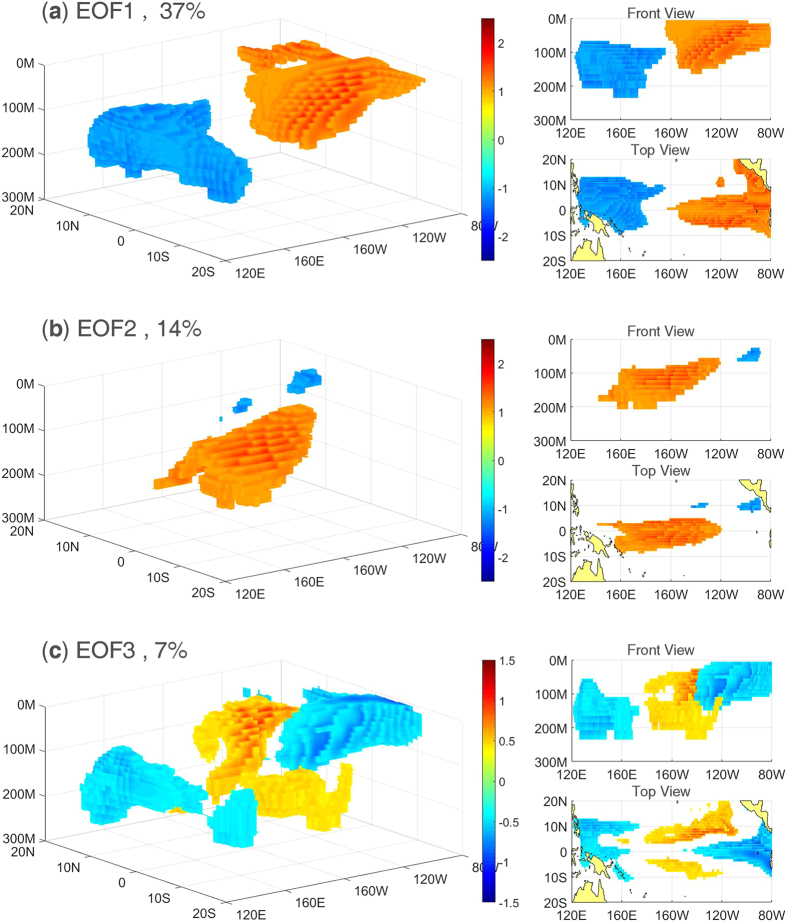
First three EOF modes of the tropical Pacific subsurface temperature anomalies from 1979 to 2014. The front and top views of each mode are shown on the right. Absolute values less than 1 (0.3) in modes 1 and 2 (mode 3) are not shown in the pictures (white regions). Unit: °C. The maps in this figure were generated using MATLAB (version: R2015b. URL link: https://www.cn.mathworks.com/products/matlab/).

**Figure 2:**
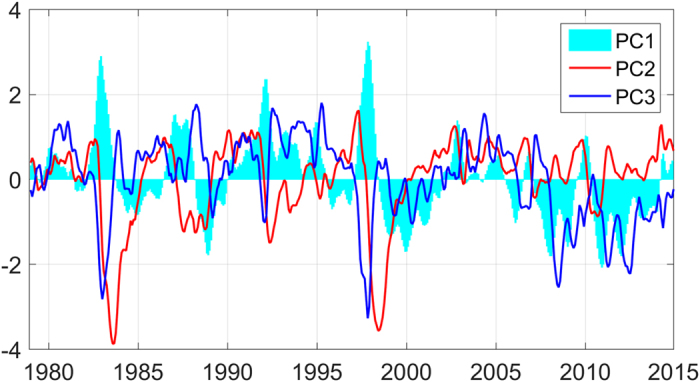
Normalized time series of PC1 (cyan bar), PC2 (red line) and PC3 (blue line) from 1979 to 2014.

The results obtained from applying EOF analysis to the tropical Pacific SOTA based on the EN4.1.1 datasets and SODA datasets are very similar. Therefore, we only present the results from the EN4.1.1 datasets (the EOF results for the SODA datasets are shown in Figures S1 and S2). According to the North criteria^16^, only the first three modes are well separated. Figure 1 shows the three leading modes. The first mode explains 37% of the total variance. As Fig. 1a shows, this mode has a dipole pattern, with warming in the eastern Pacific shallow layer and cooling in the western Pacific deep subsurface. The second mode explains 14% of the total variance and has a monopole pattern, with warming only in the central Pacific subsurface, as shown in Fig. 1b. The third mode explains 7% of the total variance and has a zonal tripole pattern, with warming in the off-equatorial central Pacific subsurface and cooling in the eastern Pacific shallow layer and western Pacific deep subsurface, as shown in Fig. 1c. Normalized time series of the three PCs are shown in Fig. 2. The skewness coefficients of the three PCs are 0.52, −1.68, −0.75, respectively, which demonstrates that mode 1 tends to appear as a positive phase while modes 2 and 3 tend to appear as negative phases. Wavelet power spectrum analysis of the three PCs shows that modes 1 and 2 each have a 2~6-yr period while mode 3 has a 1~2-yr period (not shown in figure).

The correlation coefficients between PC1, PC2, PC3 and NIÑO3 are 0.86 (much higher than the 99% confidence level of 0.54), −0.04 and −0.28, respectively, while those between the three PCs and IEMI are 0.21, 0.58 (99% confidence level of 0.46) and 0.53 (99% confidence level of 0.41), respectively. These statistical analysis results demonstrate that mode 1 is highly related to traditional El Niño while modes 2 and 3 are both related to El Niño Modoki. The time series of PC2 + PC3 has a much higher correlation (0.78, 99% confidence level of 0.43) with IEMI, which implies that El Niño Modoki generally accompanies both modes 2 and 3.

**Figure 3:**
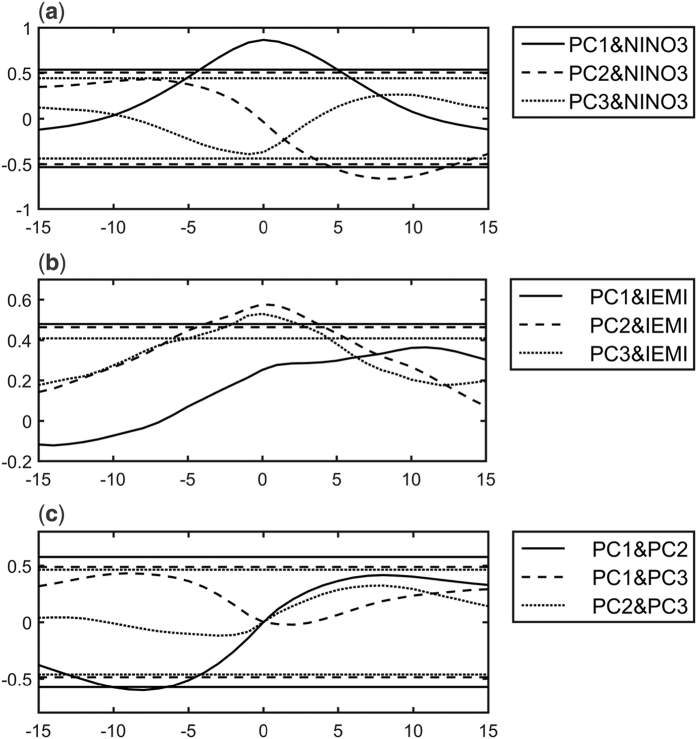
Lead-lag correlation of PC1, PC2 and PC3 with NIÑO3 (**a**) and IEMI (**b**). Lead-lag correlation between PC1, PC2, and PC3 (**c**). The straight line in each picture indicates the 99% confidence level from a two-tailed Students t-test.

The lead-lag correlations between the three PCs and NIÑO3 (IEMI) are shown in Fig. 3a,b. The maximum correlations between PC1 (PC2 and PC3) and NIÑO3 (IEMI) are at lags of 0, and no significant correlations are found between PC1 (PC3) and IEMI (NIÑO3). There is a significant maximum negative correlation when PC2 lags NIÑO3 by 8 months. Previous studies have noted that a strong La Nina Modoki often follows a strong traditional El Niño^3^,^7^,^10^. As mode 2 is related to El Niño Modoki, it is reasonable that mode 2 has a negative lag correlation with traditional El Niño. Figure 3c shows the lead-lag correlations among the three PCs. As the figure shows, PC1 has a significant negative correlation with PC2 with an 8-month lag, which indicates that mode 2 (opposite phase) tends to arise after the evolution of mode 1. PC3 has no significant lead-lag correlation with PC1 or PC2, which implies that mode 3 is relatively independent of modes 1 and 2.

**Figure 4:**
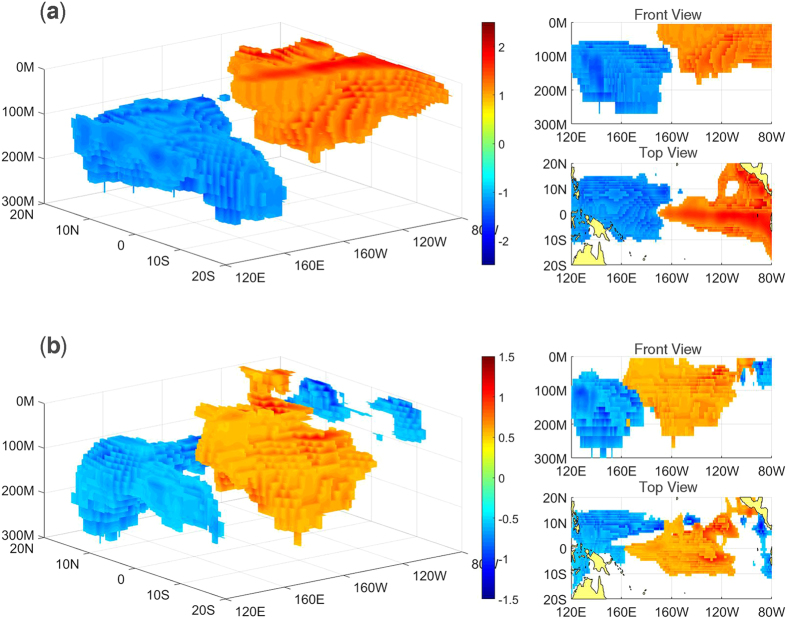
Composite SOTA distribution of five traditional El Niño events (**a**), 1982–1983, 1986–1988, 1991–1992, 1997–1998, 2009–2010) and six El Niño Modoki events (**b,** 1980–1981, 1985–1986, 1990–1991, 1994–1995, 2002–2003, 2004–2005) from May in the preceding year to May in the decaying year. Unit: °C. The regions exceeding 0.01 significance level based on the two-tailed Student’s t-test and absolute values great than 1 (0.5) in a (**b**) are shaded. The maps in this figure were generated using MATLAB (version: R2015b. URL link: https://cn.mathworks.com/products/matlab/).

Figure 4 shows the composite SOTA spatial distributions of traditional El Niño and El Niño Modoki. As the figure shows, traditional El Niño has a seesaw pattern, with negative SOTA in the western Pacific and positive SOTA in the eastern Pacific. El Niño Modoki has a zonal tripole pattern, with positive SOTA in the central Pacific and negative SOTA in the western Pacific and far eastern Pacific. Compared to the spatial distributions of the three subsurface modes (Fig. 1), mode 1 is very similar to traditional El Niño, whereas modes 2 and 3 appear to be two parts of El Niño Modoki.

**Figure 5:**
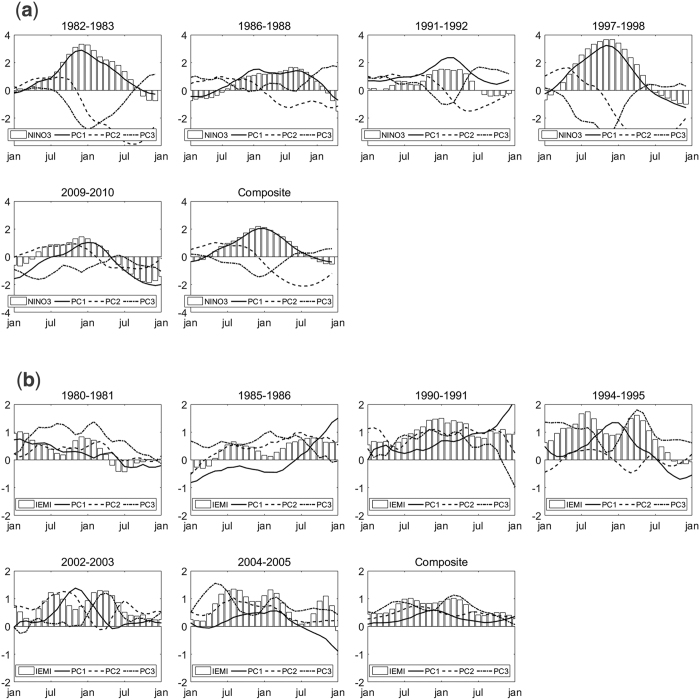
Normalized time series of PC1, PC2, and PC3 in Traditional El Niño events (**a**) and El Niño Modoki events (**b**).

To further examine the evolutions of the three modes during the two types of El Niño events, we contrast the PCs in historical El Niño events from 1979 to 2014. A traditional El Niño (El Niño Modoki) event will be identified when NIÑO3 (IEMI) exceeds 0.5 standard deviations for at least 6 months. As a result, four traditional El Niño events (1982–1983, 1986–1988, 1991–1992, and 1997–1998) and six El Niño Modoki events (1980–1981, 1985–1986, 1990–1991, 1994–1995, 2002–2003, and 2004–2005) are identified. Note that NIÑO3 and IEMI both exceed 0.5 for more than 6 months during the 2009–2010 event; therefore, the type of this event cannot be determined using this classification system at present. The normalized time series of PC1, PC2, and PC3 during the two types of El Niño are shown in Fig. 5. As the composite result (Fig. 5a) shows, the evolution of mode 1 is highly consistent with traditional El Niño. Mode 2 has an obvious phase reversal in the development and decay period and reaches a strong negative amplitude at approximately 8 months after the mature phase of El Niño. The evolutions of mode 3 are not very consistent across events but are generally in a negative phase. The 2009–2010 El Niño is hard to categorize using NIÑO3 and IEMI, as mentioned before, but the evolutions of the three modes during this event are closer to those of traditional El Niño. Thus, this El Niño event is classified as traditional El Niño. For El Niño Modoki (Fig. 5b), the composite result shows that the evolutions of modes 2 and 3 are consistent with El Niño Modoki and that mode 1 is generally in a weak positive phase. Note that the 1994–1995 event (mainly dominated by mode 3) and 2002–2003 event (dominated by modes 2, 1 and 3, in turn) are different from the composite result.

### Evolutions and possible mechanisms of the three modes

**Figure 6:**
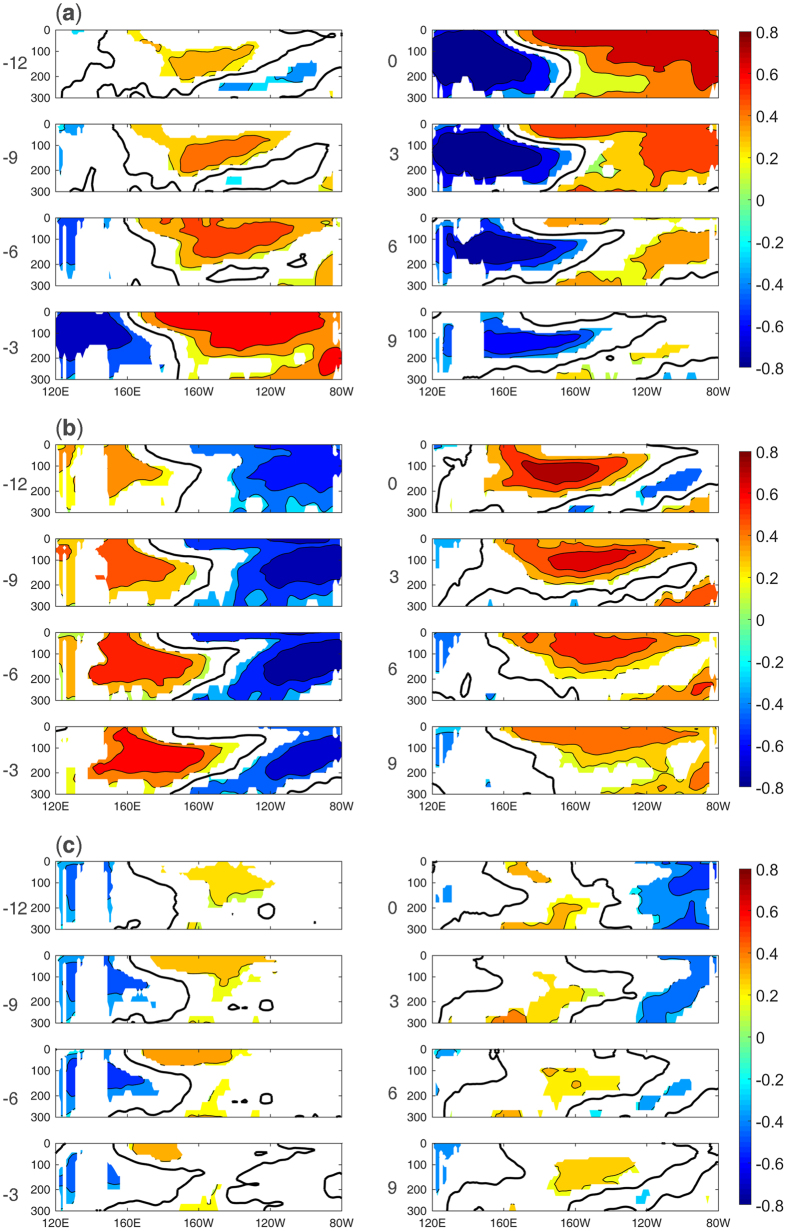
Lead-lag correlation of PC1 (**a**), PC2 (**b**) and PC3 (**c**) with SOTA averaged between 10S-10N. The black line in each box indicates the zero correlation, and the values exceeding the 90% confidence level based on a two-tailed Student’s t test are shaded. The positive (negative) number to the left of each box indicates the months that the PC leads (lags) SOTA.

In this section, we discuss the evolutions and possible mechanisms of the three subsurface modes. Figure 6 shows the lead-lag correlation between the three PCs and the SOTA averaged between 10 S and 10 N. For mode 1, the warm SOTA first appear in the central Pacific subsurface at 12 months lead, and the correlations then propagate toward the eastern Pacific along the thermocline (Fig. 6a -12~0). Next, the anomalous warming dissipates in approximately 6 months (Fig. 6a 3~9). When the positive SOTA reach the eastern Pacific, negative SOTA appear in the western Pacific subsurface (Fig. 6a -3). As the positive SOTA in the eastern Pacific decay, the negative SOTA in the western Pacific propagate eastward (Fig. 6a 3~9). Notably, the cooling does not reach the eastern Pacific but decays in the central Pacific (not shown in the figure). The evolution of mode 2 is shown in Fig. 6b. The warming first appears in the western Pacific subsurface and then propagates toward the central Pacific (Fig. 6b -12~0). Next, the propagation appears to become “blocked”. The warming continues to spread eastward, but the centre stays and decays in the central Pacific (Fig. 6b 0~9). Figure 6c shows the evolution of mode 3. The warming emerges from the central Pacific and then propagates westward in the shallow layer of the central Pacific (Fig. 6c -12~0). At the same time, cold anomalies appear in the western Pacific. After the mature phase, the cold anomalies in the western Pacific disappear, and another cooling occurs in the eastern Pacific (Fig. 6c 0~6).

**Figure 7:**
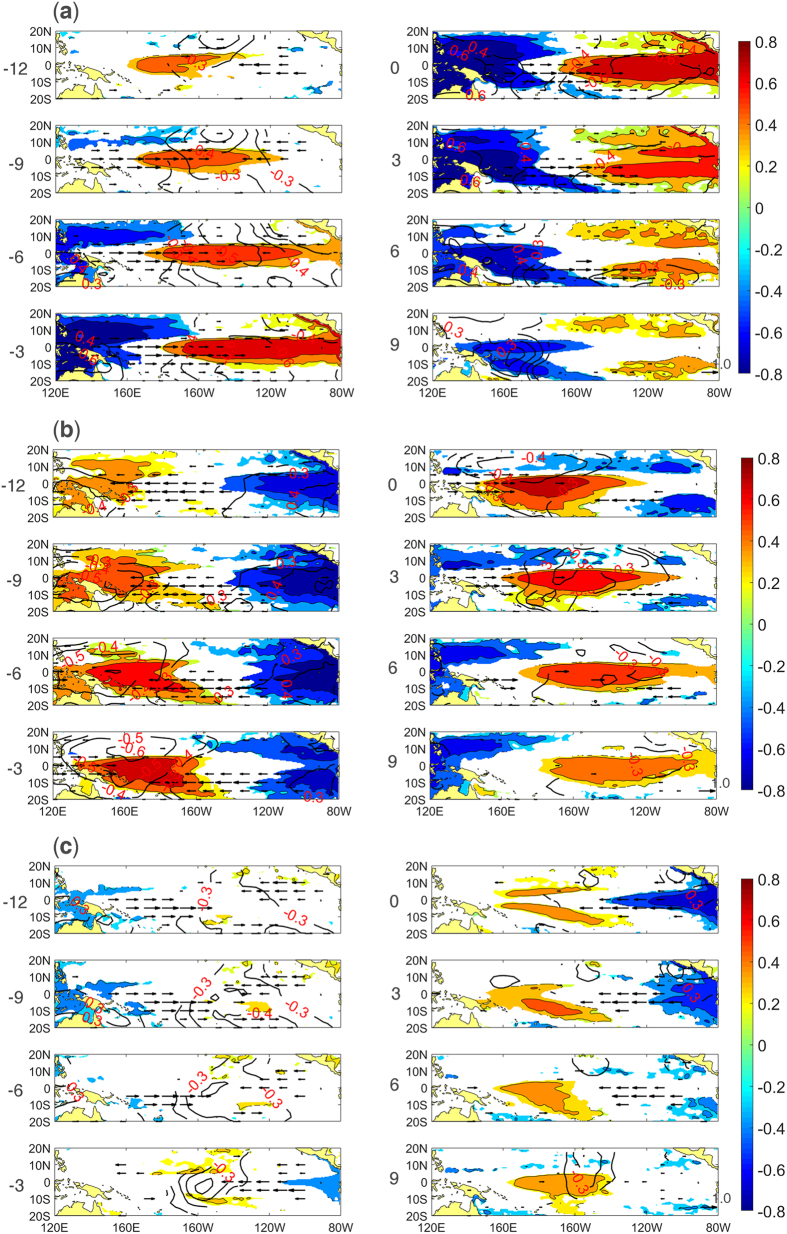
Lead-Lag correlation of PC1 (**a**), PC2 (**b**) and PC3 (**c**) with SSHA (shaded), UWNDA (vector), and SLPA (contour). The shaded areas exceed the 90% confidence level, and the vector and contour areas exceed the 99% confidence level, both based on a two-tailed Student’s t-test. The positive (negative) number to the left of each box indicates the months that the PC leads (lags) SSHA/UWNDA/SLPA. The maps in this figure were generated using MATLAB (version: R2015b. URL link: https://cn.mathworks.com/products/matlab/).

**Figure 8:**
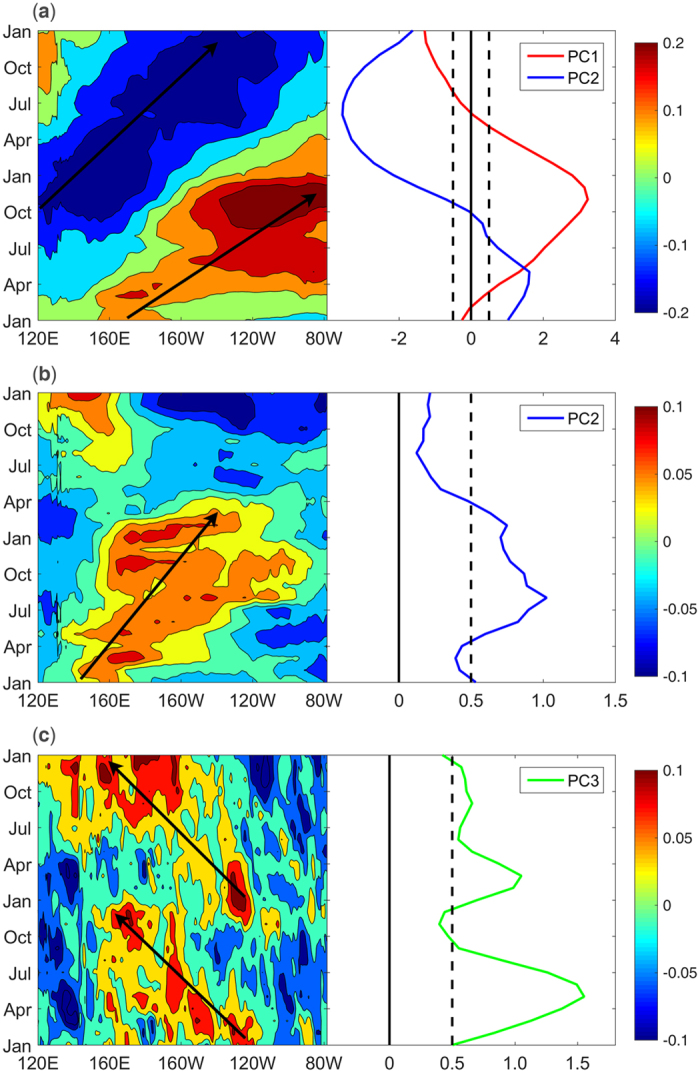
Time-longitude evolution of SSHA (left) and the corresponding PCs (right), (**a**) the equator section for the 1997–1998 traditional El Niño event, and (**b**) the equator section and (**c**) (10S + 10N)/2 section for the 2004–2005 El Niño Modoki event. The black arrows indicate the oceanic waves related to each mode. Unit: m.

Figure 7 shows the lead-lag correlation between the three PCs and the sea level height anomalies (SSHA), zonal wind anomalies (UWNDA), and sea level pressure anomalies (SLPA), respectively. Mode 1 is linked with a strong downwelling equatorial Kelvin wave that propagates from the central to the eastern Pacific, accompanied by anomalous westerlies that propagate from the western to the central Pacific within 12 months (Fig. 7a -12~0). Then, this Kelvin wave reflects as off-equatorial Rossby waves at the eastern Pacific boundary, while the anomalous westerlies gradually subside (Fig. 7a 0~9). Negative SLPA first appear in the central Pacific at 12 months lead and then move to the eastern Pacific, and positive SLPA are found in the western Pacific. Mode 2 is also linked with the equatorial Kelvin wave. However, unlike the Kelvin wave related to mode 1, this Kelvin wave propagates from the western to the central Pacific. As Fig. 7b shows, positive SSHA appear in the western Pacific and propagate toward the central Pacific (Fig. 7b -12~9). During the development period of this mode, strong anomalous easterlies exist over the central Pacific that strengthen the positive SSHA in the western Pacific (Fig. 7b -12~-3). Next, the anomalous easterlies become very weak while significant westerlies appear in the western Pacific (Fig. 7b -3~6). As a result, the SSHA begin to propagate eastward, but the anomalous centre decays in the central Pacific before reaching the eastern Pacific (Fig. 7b -3~9). Positive and negative SLPA are found in the eastern and western Pacific, respectively, during the development period. As the SSHA propagate toward the central Pacific, positive SLPA disappear, and negative SLPA move toward the central Pacific. Figure 8c shows that mode 3 is linked with a Rossby wave. The positive SSHA appear in the 160 W~120 W off-equatorial region and propagate westward to finally reach 160E~160 W (Fig. 7c -6~3). Next, the anomalies converge at New Guinea and propagate eastward along the equator (Fig. 7c 3~9). Before the propagation of the SSHA, anomalous westerlies and easterlies on the sides of the central Pacific induce the positive SSHA in the central Pacific (Fig. 7c -12~-6). At 3 months lead, the westerlies become weak, and the easterlies become strong. Due to the easterlies, the SSHA propagate toward the west to converge and reflect at New Guinea (Fig. 7c -3~6). Negative SLPA are found in the central Pacific because of the convergence of the zonal anomalous wind during the development period (Fig. 7c -12~-6). Then, the negative SLPA become weak, and positive SLPA appear in the eastern Pacific that strengthen the easterlies and further cause the westward propagation of the SSHA (Fig. 7c -3~3).

To examine the physical existence of the oceanic waves related to the three modes, we analyse the evolution of SSHA in the 1997–1998 and 2004–2005 events which have been the strongest traditional El Niño and El Niño Modoki events, respectively, since 1979. As Fig. 8a shows, during the 1997–1998 event, a downwelling Kelvin wave, which propagates from the central to the eastern Pacific, is observed in the developing and mature period (Jan 1997-Dec 1997). Next, an upwelling Kelvin wave is observed propagating from the western to the central Pacific. The evolutions of these two oceanic waves are well characterized by PC1 and PC2 shown on the right, respectively, which demonstrates a relationship to modes 1 and 2, respectively. We also noticed that mode 2 (negative phase) appeared after the El Niño had decayed, which implies that traditional El Niño might have a lead relationship with mode 2, which will be further discussed in the next section. Figure 7b,c show the equator section and average of the 10S and 10N ((10 S+ 10N)/2) section, respectively, of the 2004–2005 El Niño event. A downwelling Kelvin wave, which propagates from the western to the central Pacific (Fig. 8b), is observed in the developing and mature period (Jan 2004-Mar 2005). A downwelling Rossby wave is observed twice, in the developing and decaying periods (Jan 2004-Jan 2005 and Jan 2005-Jan 2006), respectively. As expected, PC2 and PC3, respectively, capture the two oceanic waves well.

**Figure 9:**
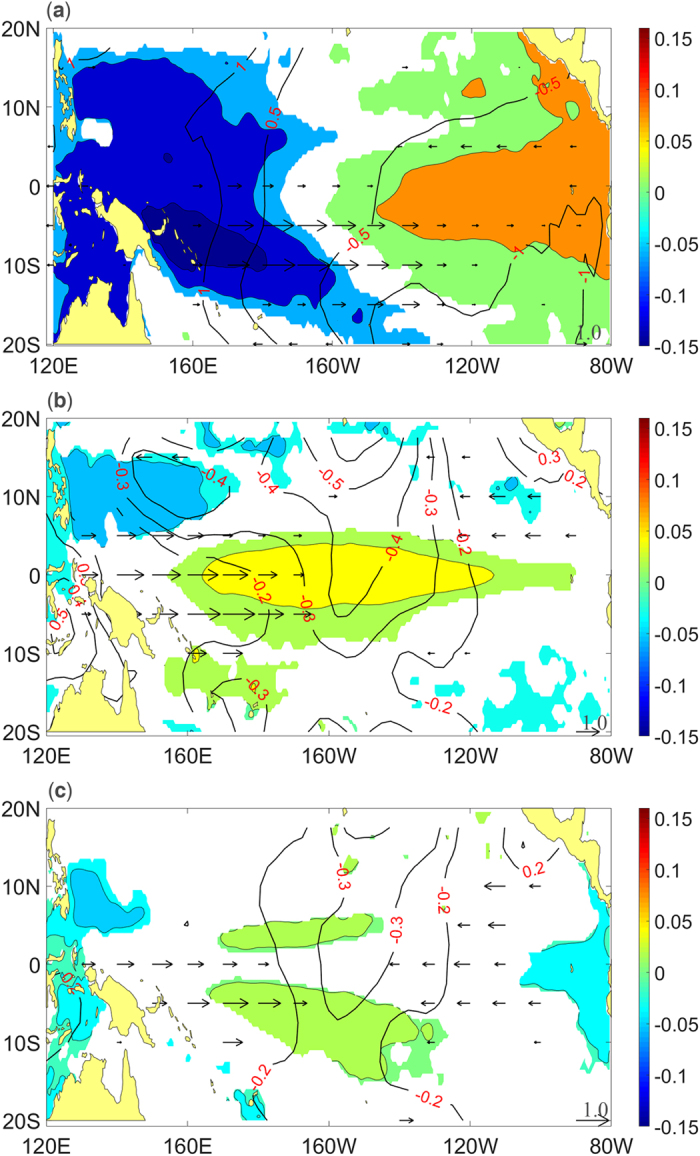
Composite distributions of SSHA (shaded, unit: m), UWNDA (vector, unit: m/s), and SLPA (contour, unit: mbar) of the events of mode 1 (**a**), mode 2 (**b**), and mode 3 (**c**). The areas shown in the picture all exceeded the 0.01 significance level based on a two-tailed Student’s t-test. The maps in this figure were generated using MATLAB (version: R2015b. URL link: https://cn.mathworks.com/products/matlab/).

The specific events of three modes are determined by their PCs from 1979 to 2014 (see Table S1). The classification standard states that a mode 1 (mode 2/mode 3) event will be determined when PC1 (PC2/PC3) exceeds 0.5 for at least 6 months. As a result, 4 (10/8) events are determined as mode 1 (mode 2/mode 3) events. The composite distributions of the SSHA, UWNDA, and SLPA of the three modes events are shown in Fig. 9. For mode 1, the SSHA and SLPA have a distinct seesaw pattern that is located in western and eastern Pacific, accompanied by strong anomalous westerlies over the central Pacific. For mode 2, positive SSHA are mainly concentrated in the central Pacific, with anomalous westerlies in the western Pacific. Positive and Negative SLPA are located in the far eastern and central Pacific, respectively. During the mode 3 event, positive SSHA are located in the off-equatorial central Pacific, and negative SSHA are located in the far western and far eastern Pacific. Negative SLPA are mainly found in the central Pacific, with anomalous westerlies and easterlies on the two sides.

## Summary and Discussion

In this study, we have identified three leading modes in the tropical Pacific SOTA using the EOF method with SOTA. The first mode has a dipole pattern, with warming in the eastern Pacific shallow subsurface and cooling in the western Pacific deep subsurface. The second mode has a monopole pattern, with only warming in the central Pacific. The third mode has a zonal tripole pattern, with warming in the off-equatorial central Pacific and cooling in the far eastern Pacific and western Pacific. Lead-lag correlation analysis and composite analysis demonstrate that mode 1 is highly related to traditional El Niño, while modes 2 and 3 are both related to El Niño Modoki.

The evolutions and possible mechanisms of the three modes are demonstrated by lead-lag correlation analysis between the variations (SOTA, SSHA, UWNDA and SLPA) and three modes. Mode 1 is linked with an oceanic Kelvin wave that propagates from the central to the eastern Pacific, which is induced by the anomalous westerlies propagating from the western to the central Pacific. Mode 2 is also linked with a Kelvin wave, but unlike in mode 1, the wave propagates from the western to the central Pacific. Significant anomalous easterlies over the central Pacific strengthen the positive SSHA in the western Pacific during the development period. As the easterlies decay and new westerlies arise over the western Pacific, accumulated SSHA begin to propagate toward the central Pacific. Mode 3 is linked with a Rossby wave that propagates from the central to the western Pacific. Mode 1 is generated by a Kelvin wave that propagates from the central to the eastern Pacific. Mode 2 is also generated by a Kelvin wave, but one that propagates from the western to the central Pacific. Mode 3 is generated by a Rossby wave that propagates westward in the central Pacific. Strong zonal convergence over the central Pacific is observed during the development period, and positive SSHA begin to propagate toward the western Pacific because of the decaying westerlies and strengthening easterlies.

Previous studies have generally considered that El Niño Modoki has an isolated pattern with variations located in the central Pacific. However, as our results have demonstrated, modes 2 and 3 are both related to El Niño Modoki and also have unique generation causes and evolutions that are different from those of El Niño Modoki, which indicates that El Niño Modoki does not have a single pattern. As mentioned previously, the evolutions of El Niño Modoki are not stable^12^, and the evolutions of modes 2 and 3 are different during certain El Niño Modoki events (Fig. 5). The findings of this research could be useful in explaining this unstable evolution feature of El Niño Modoki, which needs further research.

## Method

### Data

The monthly subsurface ocean temperature (SOT) data were obtained from the Met Office Hadley Centre observation datasets: EN4, quality-controlled SOT and salinity profiles and objective analyses^17^. The data version number used in this study is EN4.1.1, and we downloaded the data on 8 August 2015. The data are available from 1900 until present, with 1° × 1° horizontal resolution and 42 levels in the vertical direction from 5 m to 5350 m. In this paper, we chose a time period from Jan 1979 to Dec 2014 to avoid the possible influence of the climate shift in 1976^18^,^19^. We selected a depth from 5 m-300 m (20 levels) to properly contain the thermocline. The Simple Ocean Data Assimilation Reanalysis dataset^20^ (SODA version 2.0.2) was also used to examine the subsurface modes as the secondary SOT data. The monthly sea surface temperature (SST) data used in this study were obtained from the 1° × 1° Met Office Hadley Centre observation datasets: HadISST^21^ (downloaded from the website at http://www.metoffice.gov.uk/hadobs/hadisst/data/download.html). The monthly mean sea surface height (SSH) data used in this research were obtained from the SODA datasets (version 2.0.2), and the monthly mean zonal wind and sea level pressure data^22^ were from the NCEP reanalysis-derived data provided by the NOAA/OAR/ESRL PSD (Boulder, Colorado, USA) on their website at http://www.esrl.noaa.gov/psd/.

### Statistical analysis statement

The confidence levels for the correlation analysis were calculated by a two-tailed Student’s t-test. The n value for the correlation analysis in our research was calculated by the following equation:


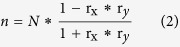


N is the length of the monthly time scale. R_x_ and r_y_ are the autocorrelation coefficients of the time series X and Y, respectively.

## Additional Information

**How to cite this article**: Zhang, Z. *et al*. Leading modes of tropical Pacific subsurface ocean temperature and associations with two types of El Niño. *Sci. Rep.* **7**, 42371; doi: 10.1038/srep42371 (2017).

**Publisher's note:** Springer Nature remains neutral with regard to jurisdictional claims in published maps and institutional affiliations.

## Supplementary Material

Supplementary Information
