# Supplementary material for: Leading modes of tropical Pacific subsurface ocean temperature and associations with two types of El Niño
Source: Sci Rep. 2017 Feb 17;7:42371. doi: 10.1038/srep42371 (PMC5314456; doi:10.1038/srep42371)
Supplement: Supplementary Information [file srep42371-s1.pdf]

Leading modes of tropical Pacific subsurface ocean temperature and associations with two types of El Niño

Zhiyuan Zhang, Baohua Ren*, and Jianqiu Zheng

Corresponding author: Baohua Ren, School of Earth and Space Sciences, University of Science and Technology of China, No.96 Jinzhai Road, Hefei, China. (ren@ustc.edu.cn)

School of Earth and Space Sciences, University of Science and Technology of China, Hefei, Anhui, China

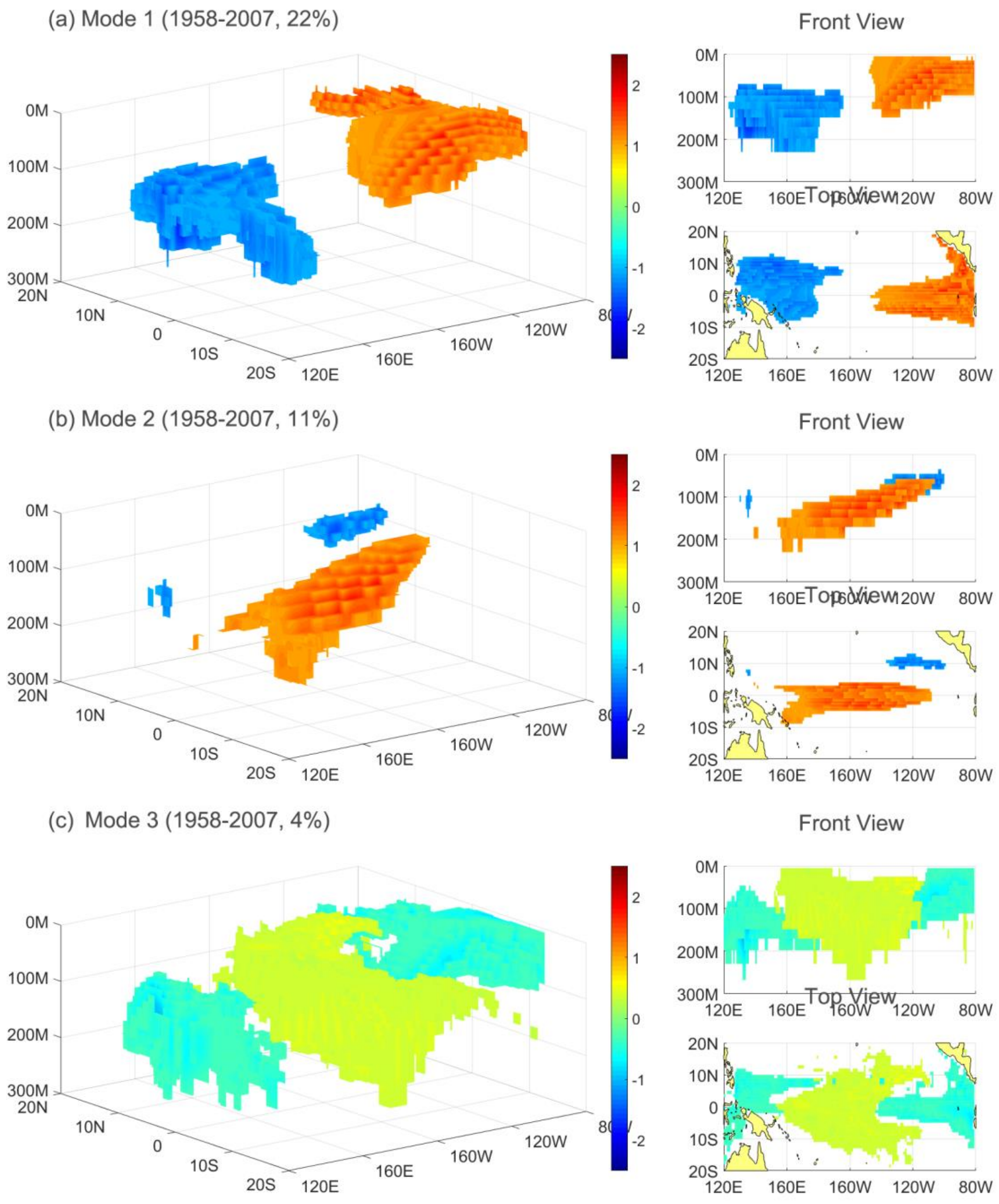

Figure S1. First three EOF modes of tropical Pacific subsurface temperature anomalies from 1958 to 2007 of SODA datasets. The front and top view of each mode are shown on the right. Absolute values less than 1 (0.3) in mode 1 and mode 2 (mode 3) are not shown in picture (white regions). Unit: ℃. The maps in this figure are generated by the MATLAB (version: R2015b. URL link: https://cn.mathworks.com/products/matlab/).

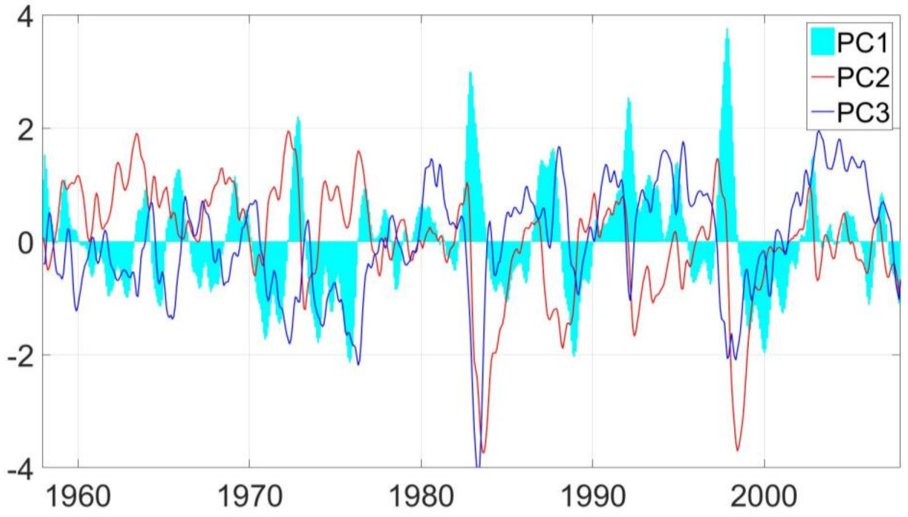

Figure S2. Normalized time series of PC1 (cyan bar), PC2 (red line) and PC3 (blue line) from SODA datasets from 1958-2007.

Table S1. Events of Mode 1, Mode 2, and mode 3 in the period from 1979 to 2014. a mode 1 (mode 2/ mode 3) event will be determined when PC1 (PC2/ PC3) exceeds 0.5 for at least 6 months. As a result, 4 (10/ 8) events are determined as mode 1 (mode 2/ mode 3) events

No.	Mode 1 events (4)	Mode 2 events (10)	Mode 3 events (8)
1	1982.9-1983.7(TE)	1982.3-1982.10(Norm)	1980.3-1981.8(EM)
2	1987.1-1988.1(TE)	1985.9-1987.2(EM)	1985.1-1986.8(EM)
3	1991.9-1992.7(TE)	1989.4-1990.4(Norm)	1987.10-1988.10(Norm)
4	1997.6-1998.5(TE)	1990.7-1991.11(EM)	1990.4-1991.8(EM)
5	2009.11-2010.4(TE)	1996.12-1997.8(Norm)	1992.6-1995.12(EM)
6		2002.1-2003.1(EM)	2003.1-2003.7/(EM)
7		2004.4-2005.4(EM)	2004.1-2005.12(EM)
8		2006.4-2006.12(Norm)	2007.2-2007.8(Norm)
9		2009.4-2009.12(TE)	
